# Targeting the immunity-cell death axis: a multi-parametric network approach of traditional Chinese medicine against ischemia-reperfusion acute kidney injury

**DOI:** 10.3389/fimmu.2026.1976812

**Published:** 2026-10-19

**Authors:** Anqi Tang, Mingquan Li, Yudi Zhang, Kaiyue Tan

**Affiliations:** 1 School of Integrative Medicine, Chongqing College of Traditional Chinese Medicine, Chongqing, China; 2 Department of Traditional Chinese Medicine, Health Center of Jinfeng Town, High-Tech Zone, Chongqing, China; 3 Department of Nephrology, Hospital of Chengdu University of Traditional Chinese Medicine, Chongqing, China; 4 The First Affiliated Hospital of Chongqing College of Traditional Chinese Medicine, Chongqing, China

**Keywords:** acute kidney injury, ischemia/reperfusion, molecular mechanisms, programmed cell death, traditional Chinese medicine

## Abstract

Acute kidney injury (AKI) remains a major clinical challenge, with persistently high incidence and mortality. Ischemia/reperfusion induced AKI (I/R-AKI) is the most common type, and its core pathological event is injury to renal tubular epithelial cells (RTECs). Energy depletion during ischemia and the burst of reactive oxygen species (ROS) during reperfusion induce metabolic reprogramming. These changes create a metabolic stress microenvironment and drive innate immune activation. This cascade begins with the release of damage-associated molecular patterns (DAMPs) and centers on sensing by pattern recognition receptors (PRRs). Macrophages then polarize toward the M1 phenotype and amplify inflammation, thereby constituting the “immunity–cell death axis.” During this process, multiple modalities of programmed cell death (PCD) interact with one another. These include apoptosis, necroptosis, pyroptosis, ferroptosis, and autophagy with dual roles. Together, they form a closely interconnected “death network,” which serves as the effector endpoint of the immune response described above. Given the extensive crosstalk within this network, interventions directed at a single death pathway have limited efficacy. Multitarget strategies that simultaneously address several key nodes are therefore emerging as a new research direction. On this basis, the present review systematically elucidates the molecular pathways of PCD in I/R-AKI and their immunoregulatory mechanisms. It also summarizes the potential mechanisms and recent advances of traditional Chinese medicine (TCM) in intervening in I/R-AKI through multipathway regulation of PCD. We hope this review provides a reference for further exploration and clinical translation in this field.

## 1 Introduction

Acute kidney injury (AKI) is a common and critical clinical condition characterized by a rapid decline in renal function over a short period, accompanied by high incidence and mortality (1). A global meta-analysis has shown that the incidence of AKI in adults reaches 21.6%, with an associated mortality rate of 23.9% (2). In China, the incidence of hospital-acquired and community-acquired AKI is 2.5% and 9.1%, respectively, with an in-hospital mortality rate of 8.8%. However, due to frequent clinical underdiagnosis, insufficient treatment, and the expanding elderly population, the true burden of AKI may be substantially underestimated (3–5). Ischemia/reperfusion induced AKI (I/R-AKI), a predominant phenotype of AKI, is characterized by a complex pathophysiology. This process involves ischemic metabolic stress and ionic dyshomeostasis, followed by reperfusion-triggered reactive oxygen species (ROS) burst, calcium overload, and inflammatory cascades. Collectively, these events activate programmed cell death (PCD) pathways, culminating in the “secondary insult” that defines renal tissue damage. Programmed cell death, including apoptosis, ferroptosis, and pyroptosis, represents a central mechanism underlying renal tubular epithelial cell injury in I/R-AKI. Single-cell sequencing studies have confirmed that renal tubular epithelial cells, due to their high metabolic activity, are particularly sensitive to ischemia and hypoxia and are more prone to undergoing PCD (6). Notably, different forms of PCD do not exist independently but interact to form a network, generating a hybrid mode of cell death that collectively regulates the progression of renal injury. Currently, effective clinical therapies targeting PCD for the treatment of I/R-AKI remain limited, highlighting the urgent need to explore novel therapeutic strategies.

Despite advances in preclinical research targeting PCD, effective clinical interventions remain lacking, largely because blockade of a single pathway is frequently undermined by compensatory signaling and crosstalk among the remaining pathways. This underscores the need for therapeutic strategies that engage multiple PCD pathways simultaneously and disrupt their interactive network. Traditional Chinese medicine (TCM) and its bioactive constituents can modulate apoptosis, necroptosis, pyroptosis, autophagy, and ferroptosis through multiple pathways, thereby offering a highly promising strategy for I/R-AKI. In the existing literature, the recent review by Wang et al. systematically catalogued the protective effects and principal signaling pathways of bioactive natural compounds such as ligustrazine, isorhamnetin, and quercetin in AKI of diverse etiologies, providing a valuable compound compendium (7). However, a compound-centered, parallel narrative fails to reveal the principles governing crosstalk among the individual death pathways, nor does it capture how the metabolic state dictates the selection of a given death modality. Accordingly, this review adopts the “immunity–cell death” axis as its organizing framework, dissects the crosstalk among distinct death modalities, and elucidates the mechanisms by which TCM constituents intervene in this intersecting network, with the aim of informing the optimization of therapeutic strategies for I/R-AKI.

## 2 Renal I/R injury and innate immunity

In I/R-AKI, acute inflammation is not initiated by exogenous pathogens but by endogenous danger signals, namely damage-associated molecular patterns (DAMPs). These DAMPs originate mainly from PCD. Modalities such as necrosis, pyroptosis, and ferroptosis disrupt the plasma, nuclear, and organelle membranes, and the resulting release of intracellular contents constitutes the principal source of DAMPs (8). DAMP release is therefore tightly coupled to PCD. Renal tubular epithelial cells (RTECs) are highly susceptible to ischemic injury, and DAMPs released from injured RTECs activate pattern recognition receptor (PRR) signaling in adjacent viable cells (9). These cells then produce proinflammatory mediators and trigger a cascade of injurious events. The severity of cell injury determines which PCD modality is engaged. PRRs, in turn, integrate local microenvironmental cues to regulate cell fate, and their activation can itself induce PCD (10). These two elements establish an immunity–cell death positive feedback loop: “cell death → DAMP release → PRR activation → secondary cell death.” This loop constitutes the core pathological axis of I/R-AKI, and it can be interrupted at either node. Blocking the DAMP pathway with PARP inhibitors or STING inhibitors remodels the intrarenal immune microenvironment and attenuates ischemic kidney injury, confirming this axis as a viable therapeutic target (11, 12). Blocking cell death is equally effective: ferroptosis inhibitors suppress immune cell activation and markedly reduce macrophage infiltration and renal injury (13). Therefore, innate immunity in I/R-AKI is a continuous process that begins with DAMP release, centers on PRR sensing, and terminates in PCD as the effector endpoint.

Macrophages are a key amplifying module of the immunity–cell death axis. DAMPs accumulation, intercellular crosstalk, or even a high-glucose microenvironment can aggravate kidney inflammation and shift macrophages from the M2 toward the M1 phenotype (14). Metabolic reprogramming in the early reperfusion phase further drives this shift toward the proinflammatory M1 phenotype. Activated M1 macrophages then promote apoptosis of RTECs, DAMP release, and synthesis of proinflammatory mediators, thereby amplifying inflammatory kidney injury (15, 16). Kidney injury molecule 1 (KIM-1) modulates the innate immune response and curbs excessive macrophage infiltration, thereby limiting kidney injury (17). Macrophage-targeted natural products have shown promise. Oridonin ameliorates I/R-AKI injury by modulating Akt-related signaling and suppressing macrophage inflammatory responses (18). Taraxasterol protects against I/R-AKI by inhibiting oxidative stress, inflammation, and apoptosis and by reducing macrophage infiltration (19). However, other studies report a different result for recombinant human erythropoietin (rHuEPO): although it modulates macrophage function, it failed to improve the severity of I/R-AKI, functional recovery, or survival in mice. The likely explanation is that rHuEPO acts on only a single node of the immune network and leaves RTEC death—the dominant driver—untouched. It is therefore insufficient to change the overall pathological outcome (**Figure 1**).

**Figure 1:**
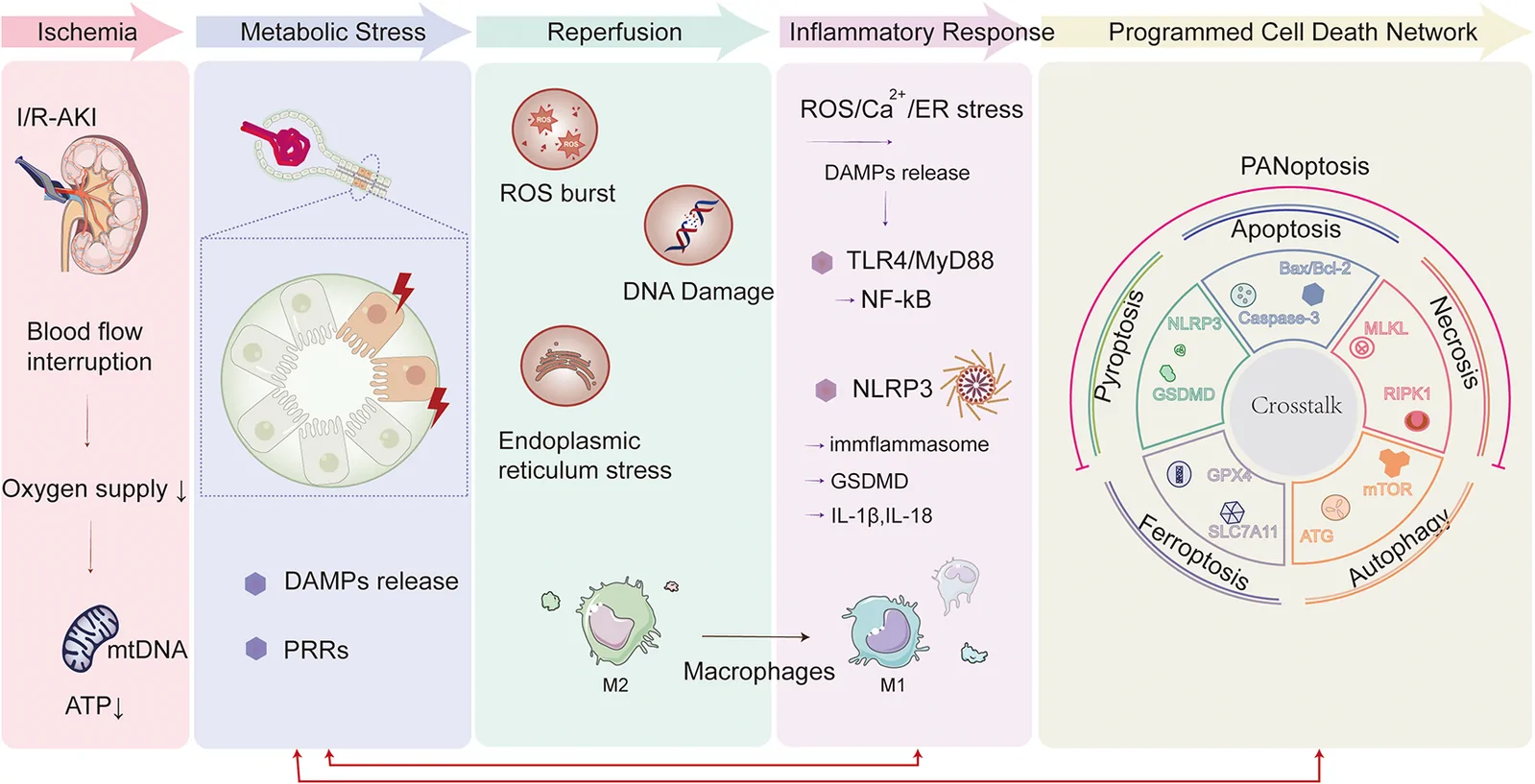
Schematic representation of the immunity–cell death axis in ischemia/reperfusion-induced acute kidney injury (I/R-AKI). Ischemia/reperfusion triggers adenosine triphosphate depletion and mitochondrial injury, resulting in the massive release of damage-associated molecular patterns (DAMPs), including mitochondrial DNA. Following recognition by pattern recognition receptors such as Toll-like receptor 4 (TLR4) and NOD-like receptor protein 3 (NLRP3), these DAMPs drive polarization of macrophages toward the classically activated (M1) phenotype and amplify the inflammatory response. Concurrently, 5 interconnected programmed cell death pathways are engaged: apoptosis, pyroptosis, necroptosis, ferroptosis, and parthanatos. The efflux of DAMPs consequent to programmed cell death, in turn, reinforces DAMP sensing and M1 polarization, thereby establishing a self-amplifying positive feedback loop. ATP, adenosine triphosphate; mtDNA, mitochondrial DNA; PRRs, pattern recognition receptors; ROS, reactive oxygen species; ER stress, endoplasmic reticulum stress; NF-κB, nuclear factor kappa-B; GSDMD, gasdermin D; IL-1β, interleukin-1β.

## 3 Apoptosis and I/R-AKI

Apoptosis is a highly regulated PCD process that plays a crucial role in I/R-AKI through intrinsic (mitochondria-mediated) and extrinsic (death receptor-mediated) pathways. During the ischemic phase, renal tissue experiences mitochondrial dysfunction due to hypoxia and insufficient nutrient supply, manifested by decreased membrane potential, restricted ATP production, accumulation of ROS, and intracellular calcium overload; these alterations increase mitochondrial outer membrane permeability, triggering cytochrome c release and activating caspase-9 and caspase-3, thereby initiating apoptosis (20). Chinese medicine components such as schisandrin B activate the Akt1 signaling pathway and regulate mitochondrial dynamics (e.g., decreasing DRP1 expression while increasing OPA1 and MFN2 expression), thereby enhancing mitochondrial resilience and suppressing apoptosis at this stage (21). In addition, although hypoxia-inducible factors under ischemic conditions can delay apoptosis by inducing mitophagy and upregulating anti-apoptotic proteins, this protective mechanism cannot completely block the overall apoptotic trend, whereas Chinese medicine intervention may enhance such endogenous protective effects (22, 23).

During the reperfusion phase, with the restoration of blood flow and oxygen supply, renal tissue is exposed to higher levels of ROS stress, accompanied by the release of large amounts of inflammatory cytokines such as TNF-α and IL-1β. These inflammatory cytokines further promote apoptosis by activating the death receptor pathway and its downstream signaling molecule caspase-8 (24). In addition, reperfusion-induced endoplasmic reticulum (ER) stress is an important trigger of apoptosis. Specifically, reperfusion-induced calcium overload exacerbates ER stress, resulting in dysregulation of the unfolded protein response. Although the unfolded protein response functions to maintain cellular homeostasis under physiological conditions, excessive or prolonged stress converts it into a pro-apoptotic signal, upregulating apoptosis-related proteins such as CHOP and inducing widespread apoptosis through both mitochondrial-dependent and independent pathways (25). Chinese medicine suppresses apoptosis through multiple mechanisms at this stage; for example, gastrodin and ligustrazine may alleviate I/R-AKI by improving oxidative damage, inflammatory responses, and apoptosis of renal tubular epithelial cells (26, 27). Salvianolic acid A exerts anti-apoptotic effects in I/R-AKI by increasing superoxide dismutase (SOD) activity, reducing MDA and ROS levels, and activating the Akt signaling pathway (28). Magnolol alleviates ER stress-related apoptosis in I/R-AKI by regulating the Akt/ERK pathway, inhibiting p38/JNK signaling, upregulating Bcl-2, and downregulating Bax and caspase-3 (29).

Pathological observations reveal plasma membrane vacuolization, cell rounding, reductions in cell and nuclear volume, and the formation of apoptotic bodies; these morphological changes confirm the occurrence of apoptosis, while Chinese medicine interventions can significantly ameliorate these structural injuries. Numerous studies have demonstrated that inhibition of ER stress can effectively reduce apoptosis and thereby alleviate I/R injury (30–32). For example, fibroblast growth factor 2 suppresses excessive ER stress and reduces I/R-induced apoptosis of renal tubular epithelial cells by activating signaling pathways such as PI3K/Akt (32). In the context of Chinese medicine, studies have shown that senkyunolide I can downregulate the expression of ER stress-related proteins such as GRP78 and CHOP (33). Meanwhile, ganoderic acids can directly inhibit the activation of caspase-8 and caspase-3. These natural compounds exert renoprotective effects by mimicking the inhibitory effects on ER stress through different mechanisms (34).

Apoptosis not only directly results in cellular loss but also interacts with other forms of cell death (such as necroptosis, pyroptosis, and autophagy), thereby aggravating renal injury through complex molecular mechanisms (35). Among these, caspase-8 is a molecular switch that determines cell death fate. However, it does not simultaneously promote multiple death modalities. Instead, depending on its catalytic activity and the composition of the signaling complex, caspase-8 channels death signaling toward a single outcome. When fully activated, it initiates apoptosis via caspase-3/7 and cleaves RIPK1/RIPK3, thereby suppressing RIPK3/MLKL-dependent necroptosis. If the necroptosis pathway is also blocked (eg, MLKL deficiency), catalytically inactive caspase-8 acts as a scaffold to activate the ASC/caspase-1 axis and redirects the death program toward pyroptosis (36). Apoptosis can mediate the orderly degradation of intracellular structures through caspase-3, and can also transform into pyroptosis via activation of the Gasdermin E pathway by caspase-3, leading to membrane rupture and activation of inflammatory responses (37). Caspase-3-deficient mice exhibit severe tubular injury in the early stage of AKI, suggesting that when apoptosis is inhibited, cells shift toward alternative death pathways such as pyroptosis or necroptosis, thereby causing more severe tissue damage (38, 39). In addition, apoptosis can inhibit autophagy by degrading autophagy-related proteins (such as Beclin-1, ATG4D, ATG3, and ATG5), thereby weakening the cellular capacity for injury repair and aggravating cellular damage (40). Chinese medicine modulates this crosstalk through multiple targets. For example, cardamonin regulates the MAPK signaling pathway, simultaneously inhibiting inflammation and apoptosis, thereby reducing the cross-activation of different forms of cell death in I/R-AKI and suggests therapeutic potential in chronic kidney injury (41). Curcumin protects the kidney and other vital organs from I/R insult through immunomodulation and inhibition of the caspase-3–mediated apoptotic pathway (42). As one of the most widely used flavonoids, naringenin protects the kidney from I/R injury by suppressing ER stress, thereby reducing both apoptosis and pyroptosis (43).

## 4 Autophagy and I/R-AKI

Autophagy is a lysosome-dependent degradation pathway that primarily degrades and recycles cytoplasmic components to help cells adapt to stress. In I/R-AKI, moderate autophagy removes damaged organelles, reduces oxidative stress, and maintains cellular energy supply, thereby alleviating renal injury (44, 45). However, excessive or prolonged autophagy may lead to autophagic cell death, aggravating renal tissue injury and even accelerating renal fibrosis in the later stages of AKI (46, 47). In addition, this lethal process differs fundamentally from the protective autophagy described above. Moreover, most reports of excessive autophagy-induced injury in I/R-AKI still lack rigorous genetic evidence. Notably, complete loss of autophagic function also exhibits complex pathological regulatory effects: a renal tubular–specific ATG7 knockout mouse model demonstrated that autophagy deficiency aggravates acute injury but significantly delays chronic fibrosis progression by suppressing the profibrotic phenotype in tubular cells (48). These findings suggest that autophagy exhibits a dynamic balance across different pathological stages of I/R-AKI. Compounds such as quercetin and scutellarein can markedly influence the pathological progression of I/R-AKI by regulating autophagy levels. Their benefit arises mainly from a moderate enhancement of protective autophagy, rather than from the induction of autophagy-dependent cell death. In the early stage of I/R-AKI, moderate activation of autophagy removes damaged mitochondria, reduces oxidative stress, and maintains energy homeostasis, thereby alleviating tubular injury. For example, quercetin activates the autophagy pathway by increasing AMPK phosphorylation and inhibiting mTOR phosphorylation, thereby exerting renoprotective effects (49). Scutellarein promotes the degradation of COX-2 protein by enhancing autophagy, thereby delaying the progression of I/R-AKI (50).

Autophagy and apoptotic signaling pathways exhibit bidirectional interactions: on the one hand, autophagy can inhibit apoptosis by suppressing the activation of apoptosis-related caspases (40); On the other hand, autophagy can also participate in ER stress-induced apoptosis: the autophagosomal membrane can serve as a signaling platform that promotes caspase-8 activation, ultimately triggering rapid apoptosis (51). Compounds derived from Chinese medicine such as Cordyceps sinensis regulate the AMPK/mTOR signaling pathway, thereby modulating autophagy intensity while inhibiting the expression of apoptosis-related proteins, reflecting their synergistic regulatory effects on intersecting pathways (52). Dihydroartemisinin alleviates renal tubular senescence by activating autophagy, thereby indirectly reducing susceptibility to apoptosis (53). This delicate interaction indicates that autophagy possesses both protective and injury-amplifying potential in I/R-AKI, and its ultimate effect depends on the intensity and duration of activation as well as the integration of microenvironmental signals. Through multi-component regulation, Chinese medicine can selectively enhance or inhibit autophagy at different pathological stages, preventing its participation in pro-apoptotic processes and thereby exerting therapeutic effects.

Mitophagy, an important subtype of autophagy, maintains cellular homeostasis by selectively eliminating damaged mitochondria, with the PINK1/Parkin pathway serving as the central regulatory mechanism. In I/R-AKI, mitochondrial depolarization in renal tubules leads to the accumulation of PINK1 on the outer mitochondrial membrane, which recruits cytosolic Parkin and triggers ubiquitination, ultimately promoting the clearance of damaged mitochondria through binding of autophagy receptors with LC3. In both *in vivo* and *in vitro* models of I/R-AKI, significant induction of mitophagy has been observed in renal proximal tubular cells, and this process is primarily regulated by the PINK1/Parkin pathway (54). When the PINK1/Parkin pathway is inhibited, damaged mitochondria cannot be effectively cleared, leading to excessive ROS production and aggravated inflammatory responses, thereby further worsening renal injury (55). Natural compounds such as quercetin enhance PINK1/Parkin pathway activity by activating SIRT1, thereby promoting mitophagy (49). In addition, the PINK1/Parkin pathway can exert additional inhibitory effects on pyroptosis by suppressing activation of the NLRP3 inflammasome, thereby further reducing inflammation and tissue injury (56). These effects collectively suggest the potential of traditional Chinese medicine in regulating the mitophagy network through multiple signaling pathways.

## 5 Ferroptosis and I/R-AKI

Ferroptosis is an iron-dependent, non-apoptotic form of PCD, whose core pathological feature is the abnormal activation of lipid peroxidation in the cellular membrane. During I/R, the oxidative stress environment generated during reperfusion synergizes with iron-dependent lipid peroxidation, ultimately triggering ferroptosis; this mechanism has been confirmed as a central pathological process in reperfusion injury (57). Notably, interventions targeting ferroptosis exert protective effects only on reperfusion injury, while showing no significant improvement in ischemic injury (58). Furthermore, clinical studies have shown that perioperative reductions in iron-binding proteins in cardiac surgery patients significantly decrease their capacity to remove catalytic iron released during cardiopulmonary bypass; this imbalance in iron homeostasis is closely associated with the occurrence of AKI, highlighting the importance of iron homeostasis in renal I/R injury (59). The application of ferroptosis-specific inhibitors (such as Ferrostatin-1) can significantly alleviate I/R-AKI, providing strong evidence supporting ferroptosis as a therapeutic target for I/R injury (60).

In the regulatory mechanism of ferroptosis, glutathione peroxidase 4 (GPX4), heat shock protein β-1, and nuclear factor erythroid 2–related factor 2 (Nrf2) constitute a key negative regulatory network that exerts cytoprotective effects by suppressing ROS biosynthesis and reducing intracellular iron levels. In contrast, acyl-CoA synthetase long-chain family member 4 (ACSL4), reduced nicotinamide adenine dinucleotide phosphate oxidase, and p53 act as positive regulators that promote ferroptosis by increasing ROS levels and inhibiting solute carrier family 7 member 11 (SLC7A11) expression (61). During the pathological progression of AKI, the antioxidant system is often functionally suppressed, with significantly decreased GPX4 activity leading to accumulation of lipid peroxidation products and subsequent acceleration of ferroptosis (62, 63). Taken together, the fate of ferroptosis depends on the balance between the positive and negative regulatory networks described above. Correcting this imbalance is therefore a key entry point for pharmacologic intervention. Specifically, Cistanche disrupts Keap1–Nrf2 binding. This increases GPX4 expression and reduces the accumulation of lipid peroxidation products in mice with I/R, thereby alleviating I/R-induced ferroptosis. Likewise, the ferroptosis-specific inhibitor ferrostatin-1 markedly improved I/R-AKI by suppressing ferroptosis (64). Total flavonoids of Desmodium styracifolium (TFDS) downregulated p53 expression and upregulated SLC7A11 and GPX4, both *in vivo* and *in vitro*. A p53 agonist abolished these effects. These findings confirm that TFDS inhibits ferroptosis and alleviates I/R by regulating the p53/SLC7A11/GPX4 signaling axis (65). In addition, vaccarin, a flavonoid glycoside extracted from the seeds of Vaccaria hispanica, attenuates ferroptosis in mice with I/R. It acts by regulating the expression of GPX4 and the cystine/glutamate antiporter (66).

Notably, these regulatory molecules do not act in isolation. Upstream sources of oxidative stress, together with metabolic and fibrotic signals, determine the intensity of lipid peroxidation. Multiple active components of TCM can target this regulatory network to intervene in kidney ferroptosis. For example, pachymic acid can inhibit renal ferroptosis by directly or indirectly activating the Nrf2 signaling pathway and upregulating the expression of downstream ferroptosis-related proteins such as GPX4, SLC7A11, and HO-1 (67). Quercetin effectively inhibits ferroptosis by reducing MDA and lipid ROS levels while increasing glutathione (GSH) content, thereby alleviating AKI (68). Salidroside inhibits I/R-induced ferroptosis by protecting renal tubular epithelial cells from oxidative damage, reducing ROS accumulation, and activating the PI3K/Akt signaling pathway (69). These effects highlight a characteristic strength of TCM in counteracting oxidative stress and limiting superoxide accumulation. Puerarin reversed the abnormal changes in SOD, MDA, GSH, and Fe^2+^ in I/R-AKI through the TLR4/Nox4 pathway. It thus achieved simultaneous inhibition of oxidative stress and ferroptosis (70). In addition, hederagenin improved tubular ferroptosis and kidney fibrosis in a diabetic kidney disease (DKD) model by inhibiting Smad3/NOX4/SLC7A11 signaling (71). These results suggest that targeting upstream fibrotic and oxidative stress signals to block tubular ferroptosis and the resulting secondary fibrosis may be an important direction for the prevention and treatment of I/R-AKI. However, the current evidence for hederagenin comes from DKD models. Its role and mechanisms in I/R-AKI remain to be verified.

There is a close molecular relationship between ferroptosis and autophagy, and specific types of autophagy, particularly selective autophagy, can directly drive the occurrence of ferroptosis. The mitochondrial dynamin-like protein OPA1 is a key regulator of mitochondrial fusion, and its dysfunction is closely associated with dysregulated mitophagy. Notably, OPA1-deficient cells exhibit significant resistance to ferroptosis, suggesting that OPA1 may enhance cellular sensitivity to ferroptosis by maintaining mitochondrial homeostasis and functional integrity. Based on these findings, QiShenYiQi dripping pills can improve mitochondrial dynamic balance and biogenesis by regulating OPA1, thereby alleviating myocardial ischemia–induced ferroptosis; whether this mechanism also applies to the improvement of ferroptosis in I/R-AKI remains to be further studied (72).

## 6 Pyroptosis and I/R-AKI

Pyroptosis, an inflammasome-mediated form of programmed cell death, plays a crucial role in I/R-AKI and is characterized by activation of inflammasomes such as NLRP3, which subsequently activate caspase-1 or caspase-11/4/5 and cleave gasdermin proteins (e.g., GSDMD), leading to cell membrane pore formation and lysis. Traditional Chinese medicine, characterized by its multi-component and multi-pathway regulation, exhibits significant potential in regulating multiple steps of the pyroptosis pathway. Puerarin, for example, suppressed NLRP3 inflammasome assembly and activation, reduced downstream caspase-1 activation and GSDMD cleavage, and decreased the release of IL-1β and IL-18 (73). These changes were associated with inhibition of the pyroptosis execution phase. In addition, it enhances the renal capacity to eliminate oxidative stress by upregulating endogenous antioxidant enzyme activity, thereby exerting dual anti-pyroptotic and antioxidant effects (73). Cordycepin inhibited NF-κB nuclear translocation and downregulated the transcription and expression of pyroptosis-related molecules such as GSDMD. These changes correlated with reduced tissue inflammation and renal injury (74). The available evidence, however, remains insufficient to establish that cordycepin alleviates pyroptosis-driven inflammation and kidney injury, and further investigation is warranted.

In I/R-AKI, the occurrence of pyroptosis is closely associated with the energy metabolic crisis of renal tubular epithelial cells (such as ATP depletion) and is jointly driven and regulated by multiple factors, including inflammatory cytokine storms, oxidative stress injury, and ER stress in the microenvironment. As a key trigger, ER stress can promote the initiation of pyroptosis by activating the NF-κB pathway and upregulating NLRP3 inflammasome expression. Gypenoside XVII significantly reduced the expression of the ER stress-related proteins BIP, p-PERK, and CHOP and downregulated BAX expression, indicating suppressed activation of apoptosis-related pathways. Levels of the pyroptosis-related proteins caspase-1 and GSDMD and of the proinflammatory cytokine IL-1β were also decreased (75). These changes were accompanied by a coordinated downregulation of pyroptosis- and apoptosis-related molecules and by attenuated renal tubular injury. The findings suggest that gypenoside XVII modulates both pyroptosis- and apoptosis-related pathways, thereby helping to confer coordinated protection of renal tubular cells.

Activation of antioxidant signaling pathways such as Nrf2/HO-1 is also an important strategy for inhibiting pyroptosis, and alleviation of oxidative stress itself can weaken the activation basis of the NLRP3 inflammasome. Naringenin and salvianolic acid B are both bioactive natural compounds and effective activators of the Nrf2 signaling pathway. Naringenin alleviates ER stress by activating the Nrf2/HO-1 axis and simultaneously suppresses ER stress-induced pyroptosis (43). Similarly, salvianolic acid B directly suppresses activation of the NLRP3 inflammasome and its downstream caspase-1/GSDMD pyroptotic execution pathway by activating the Nrf2 signaling pathway (76). Through a shared upstream target but slightly different downstream mechanisms, both compounds ultimately achieve protective effects against renal I/R injury.

Caspase-3 plays a dual role in the regulation of cell death; it is not only a key executor of apoptosis but can also induce pyroptosis by cleaving GSDME when GSDME is highly expressed. Activated caspase-3 can specifically cleave GSDME, thereby inducing pyroptosis, whereas silencing GSDME alleviates AKI and the associated inflammatory responses (77). However, blocking a single cell death pathway may have limited efficacy or even produce adverse effects—for example, GSDMD-deficient mice are more susceptible to AKI, whereas simultaneous deficiency of GSDMD and the necroptosis mediator MLKL significantly alleviates injury (78, 79). These findings indicate that inhibition of a single PCD pathway may lead to opposite outcomes, whereas simultaneous blockade of two PCD pathways can more effectively reduce tissue injury. Gypenoside XVII concurrently downregulated the expression of both pyroptosis- and apoptosis-related molecules (75). Compounds such as puerarin and salvianolic acid B inhibited pyroptosis-related pathways and exerted antioxidant effects at the same time (70, 73, 76). The capacity of TCM to coordinately regulate the apoptosis, pyroptosis, and even necroptosis networks may therefore offer broader mechanistic routes for intervention in I/R-AKI.

## 7 Necroptosis and I/R-AKI

Necroptosis is a form of programmed cell death that combines apoptotic signaling characteristics with necrotic morphological features and is considered a major cause of ischemic injury in the kidney and heart (80, 81). Unlike classical apoptosis, necroptosis is typically activated when caspase-8 activity is inhibited or absent, and its core regulatory mechanism depends on the receptor-interacting protein kinase 1/3 (RIPK1/RIPK3) signaling pathway. Specifically, formation of the RIPK1–RIPK3 complex (the necrosome) promotes phosphorylation of MLKL and its integration into the cell membrane, ultimately leading to disruption of membrane integrity and release of inflammatory cytokines, thereby triggering a pronounced inflammatory cascade. Studies have shown that significant accumulation of pMLKL can be detected in necrotic tubular regions of patients with AKI, providing the first direct evidence of necroptosis in human renal tissue (82). From the perspective of cell-type specificity, renal tubular cells and glomerular endothelial cells exhibit higher susceptibility to necroptosis compared with mesangial cells and podocytes (83).

In renal I/R, the initiation of necroptosis is closely associated with insufficient oxygen supply and nutrient deprivation during the ischemic phase. Oxidative stress and mitochondrial dysfunction induced by ischemia lead to phosphorylation and activation of RIPK1 and RIPK3, while oxidative damage and inflammatory mediator release during reperfusion further aggravate necroptotic signaling. *In vitro* studies have confirmed that necroptosis markers, including RIPK1, RIPK3, and MLKL, are upregulated in HK-2 cells subjected to H/R treatment (84, 85). Among these mechanisms, the RIPK3-dependent necroptosis pathway is considered a key mechanism leading to renal parenchymal cell necrosis during I/R (86). Genetic knockout of RIPK3 or MLKL not only effectively reduces I/R-induced necroptosis in renal tubular cells but also significantly decreases the activation of the NLRP3 inflammasome (87). Notably, interventions based on traditional Chinese medicine also target this pathological process. For example, gypenoside XLIX blocks necroptosis execution by modulating the upstream IGFBP7/IGF1R axis, and this is accompanied by suppression of the downstream RIPK1/RIPK3/MLKL signaling cascade (88). JianPiYiShen granules can improve cisplatin-induced by reducing the gene expression of RIPK3 and MLKL and downregulating key phosphorylated proteins such as P-RIPK1, P-RIPK3, and P-MLKL (89). In addition, studies have shown that 7-hydroxycoumarin (derived from Eucommia ulmoides, Prunella vulgaris, Angelica pubescens, etc.) can also alleviate renal necrosis and accelerate tissue repair by inhibiting the RIPK1/RIPK3/MLKL pathway, demonstrating protective effects against cisplatin-induced AKI; however, its exact efficacy in I/R injury requires further verification (90).

The pathological damage caused by necroptosis in renal I/R lies not only in the process itself but also in the complex network it forms with other forms of programmed cell death such as apoptosis and pyroptosis. Studies have found that MLKL and RIPK3 not only mediate necroptosis but can also trigger pyroptosis by activating the NLRP3 inflammasome, revealing cross-regulatory mechanisms among different cell death pathways (91). However, inhibiting a single pathway alone may be counterproductive; for instance, deficiency of RIPK3 or caspase-8 may upregulate intrinsic apoptosis, whereas MLKL gene knockout may lead to higher levels of apoptosis (86, 92, 93). This suggests that effective therapeutic strategies require multi-target interventions. Overall, although some progress has been made in the study of traditional Chinese medicine in regulating necroptosis, investigations into the integrated regulatory mechanisms of its complex network remain insufficient and require more systematic and in-depth research.

## 8 Multimodal coexistence and interaction of programmed cell death in I/R-AKI

During the progression of I/R-AKI, multiple cell death mechanisms coexist, and different cell death modes exhibit multi-level interconnections, including shared triggering factors, signaling molecules, and protective mechanisms. Among them, autophagy, apoptosis, and pyroptosis are typical PCD modes, which can not only promote or inhibit cell death through different pathways but may also interact with and even transform into one another under specific conditions. In the early stage of I/R, autophagy and apoptosis can exert protective cellular effects by removing damaged organelles and injury-related factors, thereby maintaining intracellular homeostasis. For example, autophagy can alleviate apoptosis by inhibiting activation of apoptosis-related caspases (94), and it can also suppress pyroptosis by reducing mitochondrial ROS levels and thereby inhibiting activation of the NLRP3 inflammasome (95). However, during sustained I/R, autophagy or apoptosis may also induce cell death under certain conditions and aggravate tissue injury. For example, the apoptotic pathway can activate GSDME through caspase-3, thereby converting apoptosis into pyroptosis (37); it may also aggravate cellular injury by degrading autophagy-related proteins and blocking the autophagic process (40). These observations indicate that autophagy, apoptosis, and pyroptosis are not isolated from one another. Instead, they form a complex and interactive regulatory network. This network can act synergistically to maintain cellular homeostasis or antagonistically to aggravate cell injury. Its final outcome depends on the characteristics of the cellular microenvironment and on the nature and intensity of the stress involved.

Certain molecules possess pleiotropic regulatory properties and can simultaneously influence multiple signaling pathways, a phenomenon known in biology as “pathway crosstalk.” This phenomenon can also be observed in I/R-AKI. For example, OPA1 is not only a regulator of mitophagy but also an important regulator of ferroptosis (96). Taking X-box binding protein 1 as another example, as an important ER stress-related transcription factor, it not only responds to ER stress by regulating the apoptotic pathway but also participates in activation of the NLRP3 inflammasome to induce pyroptosis (97, 98). In addition, MLKL and RIPK3 not only mediate necroptosis but can also trigger pyroptosis by activating the NLRP3 inflammasome (91). When multiple cell death programs are simultaneously activated, inhibition of one molecular pathway may trigger the activation of another death mechanism, thereby complicating the process of I/R injury (99). Therefore, research should not only focus on the molecular mechanisms of individual cell death pathways and their specific roles in renal injury but also employ multi-omics technologies, single-cell sequencing, and gene editing to explore the crosstalk and interconversion among different death mechanisms, thereby achieving a more comprehensive understanding of their roles in kidney injury.

PANoptosis integrates key execution components of necroptosis, apoptosis, and pyroptosis into the PANoptosome complex. It thereby enables holistic intervention in the cell death network. As a recently proposed inflammatory form of programmed cell death, PANoptosis has an integrative property. This property overcomes the limited efficacy caused by compensatory activation of other death pathways during single-pathway blockade, and it produces more robust immunomodulatory effects. Z-DNA binding protein 1 (ZBP1) functions as a cytosolic sensor of Z-form nucleic acids and serves as a pivotal regulator of PANoptosis. Through assembly of the PANoptosome, ZBP1 mediates the concurrent activation of the pyroptotic, apoptotic, and necroptotic cascades (100). Existing studies have focused largely on the involvement of PANoptosis in sepsis- and cisplatin-induced AKI (101, 102). Nevertheless, emerging evidence indicates that PANoptosis also contributes to the pathogenesis of I/R-AKI. Interferon signaling and the STING pathway cooperatively promote STAT1 activation, which exacerbates ZBP1-mediated PANoptosis and amplifies RTEC death and inflammatory injury (103). Although no natural compound has yet been shown to ameliorate I/R injury by suppressing the PANoptosis pathway, this pathway remains a promising therapeutic target. It warrants further in-depth investigation.

Numerous active compounds and herbal formulas from TCM have been shown to simultaneously regulate multiple cell death signaling pathways, thereby potentially offering broader mechanistic routes for addressing the complex pathology of I/R-AKI. For example, resveratrol attenuates apoptosis in renal I/R injury by suppressing caspase-3 activity and the downstream caspase cascade (104). In addition, restoration of autophagic activity represents a further mechanism underlying its renoprotective effect against I/R insult (105). Naringenin regulates ER stress while simultaneously inhibiting the occurrence of apoptosis and pyroptosis (43). Sanqi Oral Solution enhances autophagy levels by regulating the ERK/mTOR pathway, while simultaneously reducing apoptosis (106). In addition, Cordyceps sinensis has been found to significantly alleviate renal I/R injury by inhibiting the p38/JNK signaling pathway in renal tubular epithelial cells, with its protective effects involving the suppression of inflammation, apoptosis, and ferroptosis (107). In summary, TCM regulates PCD through multiple components and at multiple levels. This multi-mechanism combined intervention model can not only alleviate pathological injury but also provide an important complement to therapeutic strategies in modern medicine (**Figure 2**, **Table 1**).

**Figure 2:**
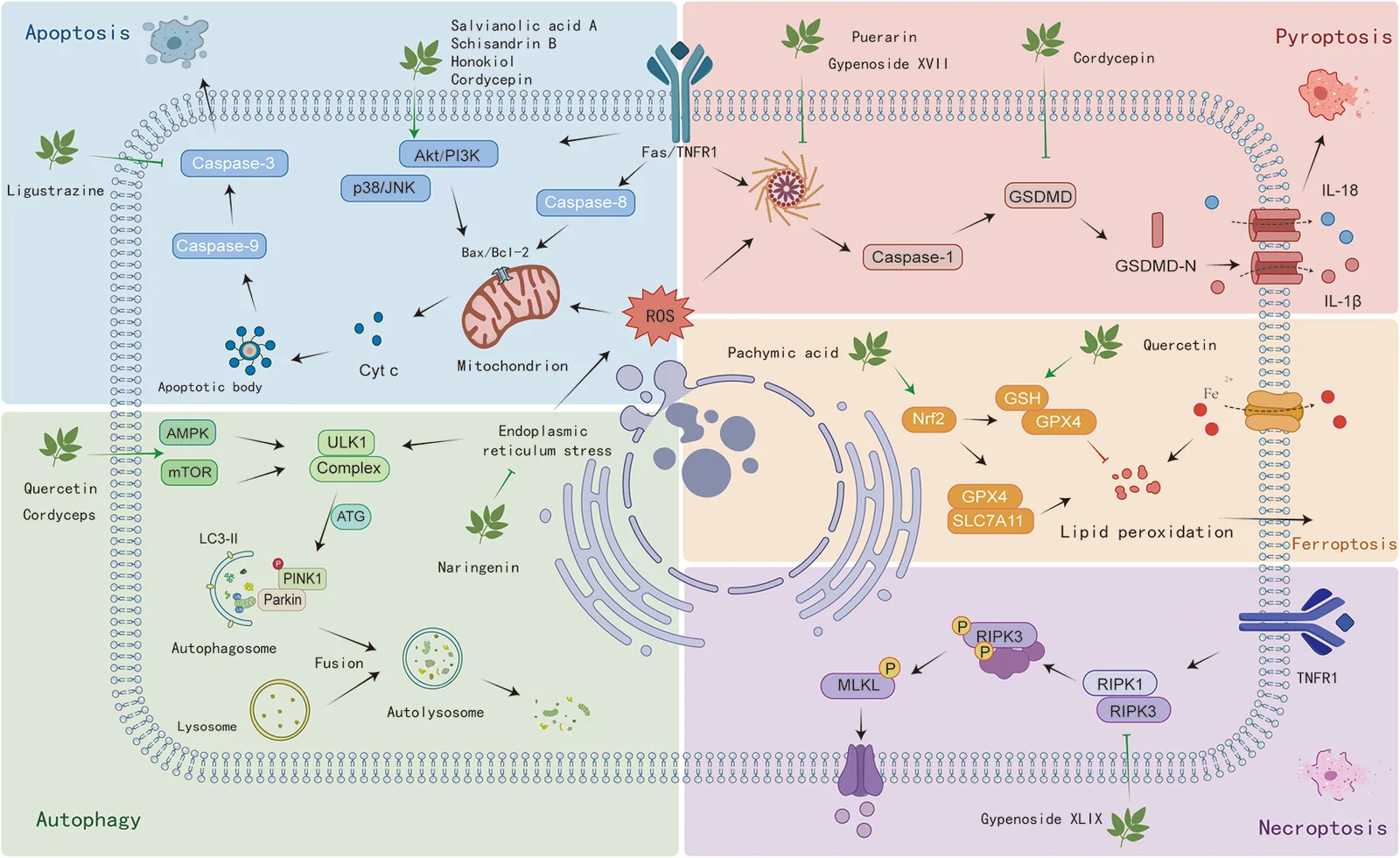
Mechanisms of natural compounds in ameliorating I/R-AKI via modulation of PCD. Green leaf icons denote natural compounds that intervene in apoptosis, pyroptosis, autophagy and necroptosis. Arrows indicate activation/promotion; blunt-ended lines indicate inhibition/suppression. I/R-AKI, ischemia/reperfusion-induced acute kidney injury; PCD, programmed cell death; ROS, reactive oxygen species; Cyt c, cytochrome c; GPX4, glutathione peroxidase 4; GSDMD, gasdermin D; IL-1β, interleukin-1β; RIPK1, receptor-interacting serine/threonine-protein kinase 1; mTOR, mechanistic target of rapamycin.

**Table 1:** Mechanisms of TCM in ameliorating I/R-AKI via modulation of PCD.

Cell death type	TCM component	Model	Mechanism	References
Apoptosis	Taraxasterol	vivo:mice, I/R; vitro:HK-2, H/R	Modulates apoptosis-related proteins (↓Bax, preserves Bcl-2), reduces macrophage infiltration.	(19)
	Schisandrin B	vivo:mice, I/R; vitro:HK-2, H/R	Activates Akt1, decreases DRP1 expression, increases OPA1 and MFN2 expression to modulate mitochondrial dynamics, and inhibits apoptosis	(21)
	Ligustrazine	vivo:rat, I/R vitro:NRK-52E cells, H/R	Reduces oxidative damage and renal tubular cell apoptosis	(26)
	Gastrodin	vivo:rat, I/R	Reduces oxidative damage and renal tubular cell apoptosis	(27)
	Salvianolic acid A	vivo:rat, I/R; vitro:HK-2, H/R	Increases SOD activity, decreases MDA and ROS levels, and activates Akt signaling to inhibit apoptosis	(28)
	Magnolol	vivo:rat, I/R	Regulates Akt/ERK, inhibits p38/JNK, modulates Bcl-2/Bax/caspase-3 to reduce ER stress-apoptosis.	(29)
	Senkyunolide I	vivo:mice, I/R; vitro:HK-2, H_2_O_2_	Decreases ER stress-related protein expression(GRP78, CHOP).	(33)
	Ganoderic acid	vivo:mice, I/R; vitro:NRK-52E, H/R	Directly inhibits caspase-8 and caspase-3 activation	(34)
	Cardamonin	vivo:mice, I/R and UUO; vitro:HK-2, H/R	Regulates MAPK to inhibit inflammation and apoptosis.	(41)
	Curcumin	vivo:rat, I/R	Inhibits caspase-3 pathway to reduce apoptosis.	(42)
	Resveratrol	vivo:rat, I/R; Vitro: NRK-52E, H_2_O_2_	Increases Nrf2 expression, decreases TLR4/NF-κB signaling, and reduces caspase-3 activity and caspase cascade activation to attenuate apoptosis.	(104)
Pyroptosis	Puerarin	vivo:mice, I/R; vitro:HK-2, H/R	Inhibits NLRP3 inflammasome assembly and activation and decreases IL-1β and IL-18 release	(73)
	Cordycepin	vivo:mice, I/R; vitro:renal macrophages,oxygen-glucose deprivation	Inhibits NF-κB activation and decreases GSDMD transcription	(74)
	Salvianolicacid B	vivo:mice, I/R; vitro:RTEC, H/R	Activates Nrf2 to inhibit NLRP3/caspase-1/GSDMD pathway.	(76)
Ferroptosis	Cistanche	vivo:mice, I/R; vitro:HK-2, H/R	Disrupts Keap1–Nrf2 binding, increases GPX4 expression, and decreases lipid peroxidation products	(64)
	Total flavonoids from Desmodium styracifolium	vivo:mice, I/R; vitro:HK-2, H/R	Regulates the p53/SLC7A11/GPX4 signaling axis and suppresses ferroptosis	(65)
	Vaccarin	vivo:mice, I/R; vitro: RTEC, H/R	Regulates GPX4 and system Xc^-^, decreases MDA and ROS levels, reduces oxidative stress, and suppresses ferroptosis	(66)
	Pachymic acid	vivo:mice, I/R	Activates Nrf2 and increases GPX4/SLC7A11/HO-1 to inhibit renal ferroptosis.	(67)
	Quercetin	vivo:mice, I/R or FA; vitro: NRK-52E and HK-2, Erastin	Decreases MDA and lipid ROS levels and increases GSH content to inhibit ferroptosis	(68)
	Salidroside	vivo:rat, I/R; vitro:NRK cell, H/R	Protects against oxidative damage, activates PI3K/Akt to inhibit ferroptosis.	(69)
	Puerarin	vivo:rat, I/R; vitro:HK-2, H/R	Modulates TLR4/Nox4 to reverse SOD/MDA/GSH/Fe^2+^ changes, inhibits oxidative stress/ferroptosis.	(70)
Autophagy	Scutellarein	vivo:rat, I/R; vitro:HK-2, oxygen glucose deprivation/reoxygenation	Enhances autophagy to promote COX-2 degradation.	(50)
Necroptosis	Gypenoside XLIX	vivo:mice, I/R or cisplatin; vitro:HK-2, cisplatin	Inhibits RIPK1/RIPK3/MLKL pathway to block necroptosis execution.	(88)
	Jianpi Yishen	vivo:mice, cisplatin;	Decreases RIPK3/MLKL expression and phosphorylation (P-RIPK1/3, P-MLKL).	(89)
	7-Hydroxycoumarin	vivo:mice, cisplatin; vitro:HK-2, cisplatin	Inhibits RIPK1/RIPK3/MLKL to alleviate necrosis and promote repair.	(90)
Multiple pathways	Naringenin	vivo:mice, I/R; vitro:HK-2, H/R	Activates Nrf2/HO-1 axis to inhibit ER stress-induced apoptosis and pyroptosis.	(43)
	Cordyceps	vivo:rat, I/R	Modulates AMPK/mTOR to regulate autophagy and inhibit apoptosis.	(52)
	Dihydroartemisinin	vivo:mice, I/R and UUO; vitro:HK-2, high-glucose-stimulated	Activates autophagy to reduce senescence and apoptosis sensitivity.	(53)
	Gypenoside XVII	vivo:mice, I/R	Inhibits the endoplasmic reticulum stress–NF-κB–NLRP3 signaling cascade to attenuate pyroptosis and apoptosis	(75)
	Resveratrol	vivo:rat, I/R; vitro:HK-2, H/R	Activates SIRT1 to enhance autophagy while reducing apoptosis.	(105)
	Sanqi Oral Solution	vivo: rat, I/R;	Regulates the ERK/mTOR pathway, reduces apoptosis, and simultaneously enhances autophagy levels.	(106)
	Cordycepin	vivo:mice, I/R; vitro:HK-2, H/R	Inhibits the p38/JNK signaling to suppress inflammation, apoptosis, and ferroptosis.	(107)

TCM, traditional Chinese medicine; I/R, ischemia/reperfusion; I/R-AKI, ischemia/reperfusion-acute kidney injury; PCD, programmed cell death; H/R, hypoxia/reoxygenation; UUO, unilateral ureteral obstruction; RTEC, renal tubular epithelial cell; FA, folic acid.

## 9 Limitations of translational research and gaps in clinical evidence

Multiple TCM preparations and their active components attenuate I/R-AKI by regulating several cell death pathways. This reflects the multipathway and holistic regulatory features of TCM. However, translation from the laboratory to the bedside still faces systemic barriers, and current studies show clear gaps at three levels, namely the dosing regimen, the material basis, and the level of evidence.

At the level of dosing regimens, many bioactive natural compounds share a common pharmacokinetic limitation. Quercetin and dihydroartemisinin, for example, generally have poor water solubility and low oral bioavailability (108, 109). Their *in vivo* exposure is therefore insufficient. Moreover, existing pharmacokinetic data come largely from healthy subjects. In AKI, however, reduced renal blood perfusion, altered plasma protein binding, and impaired clearance can markedly change drug disposition. Relevant pharmacokinetic data in this setting are especially scarce. As a result, the preclinical dose-exposure relationship is difficult to extrapolate to clinical practice. To address this bottleneck, nanodelivery systems hold promise for improving bioavailability through solubilization, prolonged circulation, and kidney-targeted delivery. They represent a formulation strategy worthy of further exploration.

Regarding dose extrapolation, allometric scaling based on body surface area has become the default tool for dose conversion between animals and humans, largely because of its operational convenience. This convenience, however, carries hidden risks. Calculation errors arising from inappropriate conversion procedures or improper selection of safety factors can directly compromise the estimation of the maximum recommended starting dose for first-in-human trials, with potentially serious consequences. Reliable dose conversion therefore cannot rely on body surface area alone. It must also integrate pharmacological characteristics, physiological and anatomical differences, pharmacokinetic parameters, metabolic function, receptor-related properties, and interspecies differences in lifespan (110).

Compounding this complexity, batch-to-batch variability in the quality of raw TCM materials and the lack of standardized extraction procedures cause the actual *in vivo* exposure in animal experiments to fluctuate. Subsequent extrapolation therefore rests on an imprecise dosing basis, and errors are amplified at each step. In addition, preclinical dosing relies mainly on intraperitoneal injection and oral gavage. These routes differ substantially from clinical intravenous or oral administration in terms of absorption, distribution, and local exposure. Moreover, solubilizing agents used in some studies, such as dimethyl sulfoxide (DMSO) and Tween surfactants, themselves possess biological activity. Their effects are bidirectional. At low concentrations, they may produce protective effects, whereas at high concentrations they become cytotoxic and can even directly alter the mode of cell death (111, 112). Such effects are sufficient to confound the interpretation of cell death outcomes. Therapeutic timing represents another key constraint. Some compounds exhibit kidney protective effects when administered before ischemia or injury (34, 42, 89). In clinical practice, however, AKI is often recognized only after injury is established, so the clinical feasibility of pretreatment is extremely limited.

At the level of material basis and safety, data on long-term toxicity, cumulative toxicity, risks of liver and kidney injury, and drug-drug interactions are largely lacking for raw TCM materials. Some components are intrinsically nephrotoxic or are excreted mainly by the kidney, and these risks are further amplified in patients with impaired kidney function. The therapeutic window and benefit-risk ratio therefore remain unknown. The geographic origin, harvest time, processing method, and extraction procedure of raw TCM materials markedly influence the constituent profile. Most studies of these materials, however, lack fingerprint profiles, content assays, and batch-consistency data. Results across different laboratories are therefore difficult to reproduce.

At the level of evidence, existing clinical evidence uniformly points to short-term benefit but lacks long-term prognostic data. A retrospective study by He et al. found that TCM decoctions containing Astragalus and Salvia were associated with short-term recovery of kidney function in patients with AKI (113). A randomized controlled trial (RCT) confirmed that the compound preparation Yiqi Peiyuan granule improved short-term outcomes in these patients (114). A systematic review likewise indicated that Shenkang injection was associated with short-term recovery of kidney function (115). Although the level of evidence increases stepwise from retrospective studies to RCTs and then to systematic reviews, the outcome measures have remained confined to short-term recovery of kidney function. Long-term prognostic data with endpoints such as progression to chronic kidney disease, need for kidney replacement therapy, or all-cause mortality are still lacking. In addition, most available clinical evidence comes from small-sample, single-center studies that rely on surrogate endpoints such as serum creatinine and urine output. Target validation data in human kidney tissue or body fluids are absent. Whether the ferroptosis, pyroptosis, and necroptosis pathways proposed in preclinical studies are predominant in human AKI has not been confirmed.

Based on these limitations, future research could focus on the following areas, which are similar to the recommendations made by Wang et al. (116). First, integrating multi-omics technologies such as transcriptomics, proteomics, and metabolomics to systematically identify key targets and biomarkers within the programmed cell death network under TCM intervention. Second, developing physiologically relevant models such as human-derived organoids or kidney-on-a-chip systems to simulate the clinical microenvironment of renal I/R injury and accurately evaluate the regulatory effects of herbal components on death pathways at a cell-type-specific level. Third, standardize dose extrapolation and strictly include matched vehicle control groups. Candidate components should also be validated in post-injury administration models, rather than in pretreatment-only models. Fourth, advance clinical translation in stages. Early-phase clinical trials, namely phase 1/2 trials, should first be conducted in patients with AKI. These trials should assess safety, pharmacokinetics, and the dose–exposure relationship. Injury biomarkers can be used for population enrichment and for preliminary validation of target regulatory activity. Once these human data are complete, multicenter RCTs can be conducted to confirm clinical efficacy. These efforts will collectively promote the translation of TCM from fundamental mechanistic research to clinical application, providing high-level evidence and novel therapeutic strategies for the prevention and treatment of AKI and related diseases.

## 10 Conclusion

I/R-AKI is an important research topic in the field of nephrology, and its pathogenesis is closely associated with PCD. PCD encompasses multiple modalities, including autophagy, apoptosis, pyroptosis, ferroptosis, necroptosis, and PANoptosis. Each modality has relatively independent signaling pathways, yet extensive crosstalk and mode switching exist among these pathways. Blockade of a single pathway often yields limited efficacy because other pathways are compensatorily activated, which further complicates the injury process. In addition, a positive feedback axis exists, namely “cell death → DAMPs release → PRRs activation → secondary cell death.” This axis suggests that the immune system serves as a central hub linking cell death and inflammation. Given its multicomponent, multitarget, and multilevel properties, TCM holds the potential to coordinately modulate both the cell death network and immune regulation. However, translation from bench to bedside remains constrained by three gaps, namely dosing regimens, the material basis, and the level of evidence. Only by giving equal weight to mechanistic research and evidence-based translation can TCM advance from basic mechanisms to clinical application. This will provide high-level evidence and new therapeutic strategies for the prevention and treatment of AKI and related kidney diseases.
